# Bronchial biopsy specimen as a surrogate for DNA methylation analysis in inoperable lung cancer

**DOI:** 10.1186/s13148-017-0432-5

**Published:** 2017-12-20

**Authors:** Sang-Won Um, Hong Kwan Kim, Yujin Kim, Bo Bin Lee, Dongho Kim, Joungho Han, Hojoong Kim, Young Mog Shim, Duk-Hwan Kim

**Affiliations:** 1 Department of Internal Medicine, Samsung Medical Center, Research Institute for Future Medicine, Sungkyunkwan University School of Medicine, Seoul, 135-710 Korea; 2 Department of Thoracic and Cardiovascular Surgery, Samsung Medical Center, Sungkyunkwan University School of Medicine, Seoul, 135-710 Korea; 3 Department of Molecular Cell Biology, Samsung Biomedical Research Institute, Sungkyunkwan University School of Medicine, Suwon, 440-746 Korea; 4 Department of Pathology, Samsung Medical Center, Sungkyunkwan University School of Medicine, Seoul, 135-710 Korea; 5 Research Institute for Future Medicine, Samsung Medical Center, #50 Ilwon-dong, Kangnam-gu, Professor Rm #5, Seoul, 135-710 Korea

**Keywords:** Hypermethylation, Lung cancer, Bronchial biopsy, Surrogate, Inoperable

## Abstract

**Background:**

This study was aimed at understanding whether bronchial biopsy specimen can be used as a surrogate for DNA methylation analysis in surgically resected lung cancer.

**Methods:**

A genome-wide methylation was analyzed in 42 surgically resected tumor tissues, 136 bronchial washing, 12 sputum, and 8 bronchial biopsy specimens using the Infinium HumanMethylation450 BeadChip, and models for prediction of lung cancer were evaluated using TCGA lung cancer data.

**Results:**

Four thousand seven hundred and twenty-six CpGs (*P* < 1.0E-07) that were highly methylated in tumor tissues were identified from 42 lung cancer patients. Ten CpGs were selected for prediction of lung cancer. Genes including the 10 CpGs were classified into three categories: (i) transcription (*HOXA9*, *SOX17*, *ZNF154*, *HOXD13*); (ii) cell signaling (*HBP1*, *SFRP1*, *VIPR2*); and (iii) adhesion (*PCDH17*, *ITGA5*, *CD34*). Three logistic regression models based on the 10 CpGs classified 897 TCGA primary lung tissues with a sensitivity of 95.0~97.8% and a specificity of 97.4~98.7%. However, the classification performance of the models was very poor in bronchial washing samples: the area under the curve (AUC) was equal to 0.72~0.78. The methylation levels of the 10 CpGs in bronchial biopsy were not significantly different from those in surgically resected tumor tissues (*P* > 0.05, Wilcoxon rank-sum test). However, their methylation levels were significantly different between paired bronchial biopsy and washing (*P* < 0.05, Wilcoxon signed-rank test).

**Conclusions:**

The present study suggests that bronchial biopsy specimen may be used as a surrogate for DNA methylation analysis in patient with inoperable lung cancer.

**Electronic supplementary material:**

The online version of this article (10.1186/s13148-017-0432-5) contains supplementary material, which is available to authorized users.

## Background

Lung cancer is the most common cause of cancer-related deaths in the world. Despite significant advances in the diagnosis and treatment of the disease, the prognosis remains extremely poor [1]. The poor prognosis results largely from occult metastatic dissemination of cancer cells nearby lymph nodes or tissues, which are found in more than half of all patients with lung cancer at the time of diagnosis, and from early recurrence after curative surgical resection. Recently, precision medicines that target potential oncogenic driver mutations have been approved to treat lung cancer [2]. However, some lung cancer patients do not have targetable mutations, and many patients develop resistance to targeted therapy. Tumor heterogeneity and mutational density are also a challenge in treating lung cancer, which underscores the need for developing alternative therapeutic strategies for treating lung cancer. Epigenetic changes involve DNA methylation, histone modification, and microRNA alteration. While oncogenic mutations in human cancer cells are irreversible, alterations in epigenetic machinery are potentially reversible, and this reversibility makes them promising therapeutic targets.

Epigenetic changes have been increasingly studied in lung cancer. These changes are observed frequently in lung cancer, and they correlate with tumor suppressor gene silencing and oncogene activation. Among epigenetic alterations, the de novo methylation of CpG islands in the promoter regions of tumor suppressor genes is usually associated with transcriptional silencing of such genes and is one of the acquired epigenetic changes that occur during the pathogenesis of lung cancer. Epigenetic therapies may circumvent the problems of tumor cell heterogeneity and drug resistance by inducing the expression of silenced tumor suppressor genes and may be more effective for lung cancer relapses that follow conventional treatment [3, 4]. In addition, there is growing emphasis on using epigenetic therapies to reprogram neoplastic cells prior to other anti-cancer therapies. To date, DNA methyltransferase (DNMT) inhibitors or histone deacetylase (HDAC) inhibitors in combination with cytotoxic agents and targeted therapies have been clinically tested in lung cancer [5–12]. Recent studies have reported that epigenetic priming agents may render tumor cells more susceptible to cytotoxic chemotherapy and molecular targeted therapy including immunotherapy. Pretreatment with epigenetic drugs prior to immune checkpoint modulators such as CTLA-4, PD-1, and PD-L1 inhibitors has shown observable responses in lung cancer patient [5, 12]; cytotoxic chemotherapy after epigenetic therapy has also shown remarkable responses in lung cancer [6].

For precision medicine using epigenetic priming prior to conventional standard therapy or targeted cancer therapy in lung cancer, analyzing clinically relevant predictive response biomarkers in lung tumor tissue is needed. However, it is difficult to obtain tumor tissues in inoperable patients with advanced lung cancer. In situations where obtaining tumor tissue is difficult, an alternative approach is to use surrogate specimens for analyzing DNA methylation in tumor tissues and to apply the results to epigenetic therapy. In this study, we analyzed if bronchial washing and bronchial biopsy specimens can be used as a surrogate for analyzing DNA methylation in surgically resected tumor tissues.

## Methods

### Study population

A total of 118 lung cancer patients and 60 healthy individuals who were admitted for curative surgical resection of lung cancer or for bronchoscopy at the Samsung Medical Center in Seoul, Korea between March 2010 and August 2016 participated in this study. One hundred and thirty-six (76 lung cancer patients and 60 hospital-based controls) bronchial washings and 42 tumor and matched normal tissues were obtained with written informed consent from all participants. Bronchoscopy in control group was performed to rule out lung cancer. Paired bronchial washing and bronchial biopsy specimens were collected only from eight (11%) of 76 lung cancer patients receiving bronchoscopy. Flexible fiberoptic bronchoscopy and sample preparation were performed as described previously [13]. Bronchial washing was performed by instilling 10 mL of sterile warm saline before obtaining biopsy samples to avoid contamination by the sloughing-off of bronchial cells during bronchial biopsy. The presence of malignant cells in the bronchial washing fluid was confirmed by cytologic examination. Individuals who were clinically free of any cancer at the time of bronchoscopy and did not have malignancy on their chest X-rays or CTs were included in the control group. The control group had tuberculosis, actinomycosis, bronchiolitis, pneumonia, or anthracofibrosis. Benign lung tumors such as hamartoma and localized organizing pneumonia were excluded in this study because their methylation profiling is not known and can lead to misclassification. This study was approved by our Institutional Review Board (IRB #: 2010-07-204) at the center. The pathologic stage was determined according to the guidelines of the tumor-node-metastasis (TNM) classification of the American Joint Committee on Cancer Staging Manual [14].

### Genome-wide methylation analysis

Genomic DNA was isolated from bronchial washing using a QIAamp DNA Blood Mini Kit (Qiagen, Valencia, CA), from sputum using the Sputum DNA Isolation Kit (Cat# 46200; Norgen Biotek, Thorold, ON, Canada) and from fresh-frozen tissue using the DNeasy Blood & Tissue kit (Qiagen) according to the manufacturers’ instructions. Bisulfite conversion was performed using the Zymo EZ DNA Methylation Kit (Zymo Research, Orange, CA), and methylation levels were measured using the Infinium HumanMethylation450 BeadChip (450K) (Illumina, San Diego, CA) according to the manufacturer’s protocol. Scanned images were processed using the GenomeStudio Methylation Module (version 2011.2). Preprocessing of 450K data was conducted using wateRmelon package in R (version 3.1.1) with Bioconductor 3.1 [15]. Methylation levels (β-values) were estimated as the ratio of signal intensity of methylated alleles to the sum of methylated and unmethylated signal intensity of the alleles. The β-values vary from 0 (no methylation) to 1 (100% methylation).

### Pyrosequencing

To validate the methylation levels from the 450K array, pyrosequencing for cg27364741 at the promoter region of *OTX1* gene was conducted using the PyroMark Q24 ID System (Qiagen) according to the manufacturer’s protocol. Biotinylated PCR primer sets for the amplification of the CpG were purchased from Qiagen (Cat no. PM00616336).

### Feature selection for lung cancer classification

Tumor-specific CpGs for lung cancer classification were selected in the following order: (i) selection of differentially methylated CpGs; (ii) removal of age-related methylation sites; (iii) gene ontology (GO) analysis (gene set enrichment analysis); (iv) feature selection based on a supervised machine learning algorithm; and (v) test of model performance. Gene ontology analysis in a set of genes was performed using DAVID (https://david.ncifcrf.gov//), and annotation clusters for which Bonferroni *P* value was below 1.0E-5 were selected as candidate GO terms for model building.

### Statistical analysis

Continuous variables were analyzed using Wilcoxon rank-sum test for independent samples or Wilcoxon signed-rank test for paired samples. Correlations between two continuous variables were analyzed using Pearson’s or Spearman’s rank correlation coefficients. Multivariate logistic regression analysis was conducted to estimate the relationship between the development of lung cancer and the CpGs found to be statistically significant in the univariate analysis. Statistical analysis was performed using R software (version 3.1.1), and supervised machine learning algorithms were carried out using RapidMiner 5.1. The performance of a model was measured using receiver operating characteristic (ROC) curves, which were created with MedCalc software.

## Results

### Preprocessing of 450K array

**Fig. 1:**
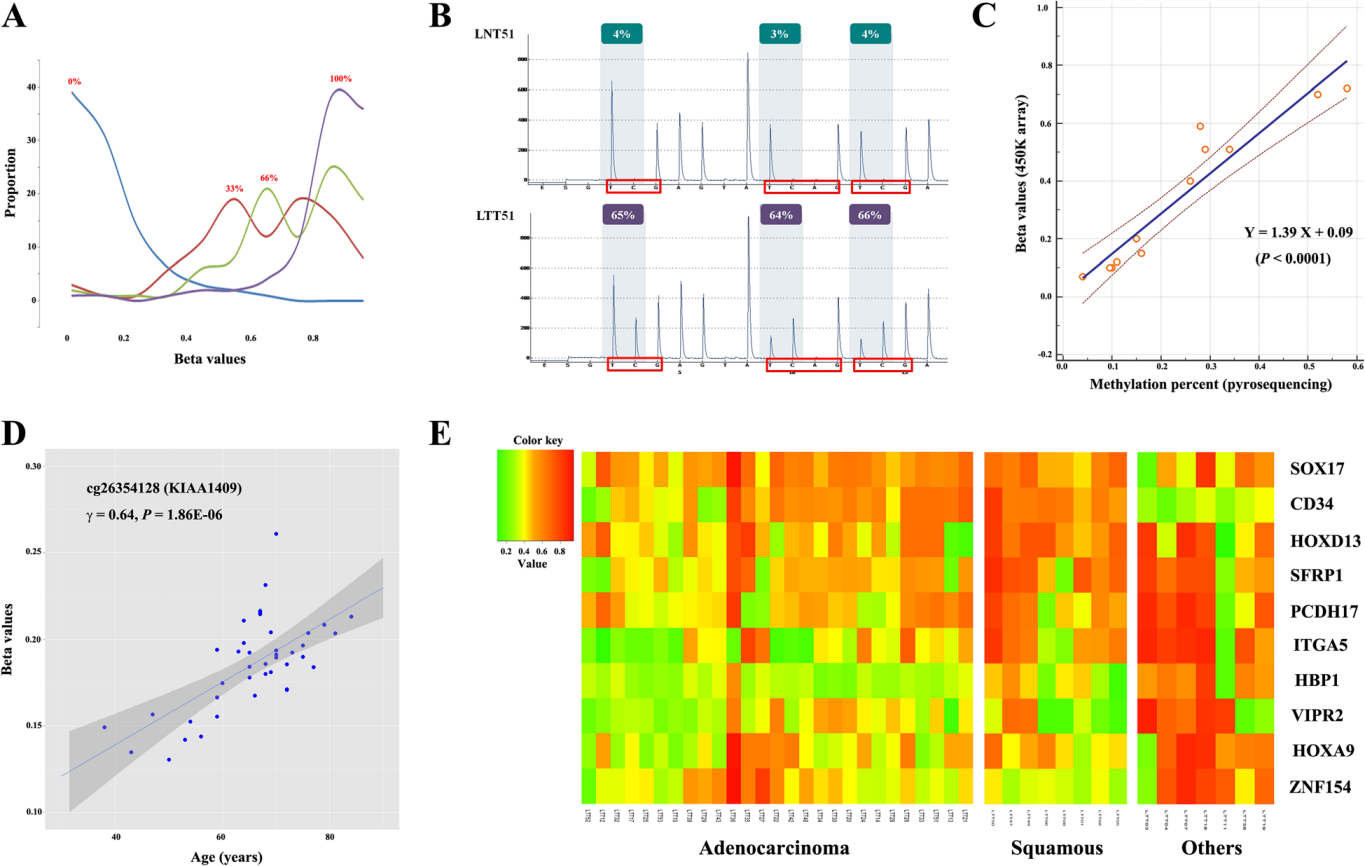
Selection of lung cancer-specific methylation. **a** The quality of the 450K array was checked by analyzing measured values for predefined subsets of methylation levels (0, 33, 66, and 100%) that were prepared by mixing fully methylated and unmethylated human control DNA. The *X*- and *Y*-axes indicate β-values of CpGs and the density of CpGs corresponding to individual β-values, respectively. **b**, **c** Methylation levels identified by the 450K array were validated using pyrosequencing. Methylation levels at a randomly selected cg27364741 locus are higher in 450K array than in pyrosequencing. **d** The relationship between patient’s age and β-values from 450K array was analyzed using Spearman’s correlation coefficient in 42 normal tissues. Methylation levels at cg26354128 loci show a linear relationship to age. **e** Methylation levels of finally selected ten genes were compared according to histology. “Squamous” indicates squamous cell carcinoma. The color spectrum from green (0%) to yellow (30%) to red (100%) indicates the levels of DNA methylation. The *X*-axis indicates the identification number of lung tumor tissue (LTT)

Clinicopathological characteristics of 118 lung cancer patients are summarized in Table 1. The assay quality of the 450K array was tested by comparing measured levels with DNA methylation levels of predefined subsets (0, 33, 66, and 100%) that were prepared by mixing fully methylated and unmethylated human control DNA (Qiagen, Hilden, Germany). The β-values in control DNA of 33% methylation were slightly inflated than true values, and β-values in control DNA of 0% methylation were approximately 0~0.3 (Fig. 1a). Pyrosequencing also suggested that β-values from the 450K array seemed to be slightly inflated than true values; for example, a cg27364741 locus at the *OTX1* gene that is known to be significantly methylated in lung cancer showed higher methylation in 450K array than in pyrosequencing, suggesting background signal of the 450K array (Figs. 1b, c). Preprocessing including type I, II bias correction was performed using the dasen function of the wateRmelon package, and 4.665 (0.97%) of 485,577 CpGs were filtered out.

**Table 1:** Clinicopathological characteristics of lung cancer patients

	Surgically resected tumor tissue	Bronchial washing
	Lung cancer (*N* = 42)	Lung cancer (*N* = 76)
Age (years)^a^	60 ± 10	59 ± 10
Sex		
Men	32	59
Women	10	17
Pack-years^a^	31 ± 29	29 ± 27
Histology		
Adenoca	27	44
Squamous	8	22
Others	7	10

*Adenoca* adenocarcinoma, *Squamous* squamous cell carcinoma

^a^Values indicate mean ± standard deviation

### Identification of differentially methylated regions

To identify differentially methylated regions (DMRs) in 42 tumor and matched normal tissues, Pearson’s Chi-square (or Fisher’s exact) test was utilized after dichotomizing the data using a β-value threshold of 0.3 because background signal in normal tissues was mostly below β = 0.3. In addition, the distribution of β-values did not follow a normal distribution; they were negatively skewed, especially in tumor tissues. Four thousand seven hundred and twenty-six DMRs for which the *P* value was less than or equal to 1.0E-07 (Bonferroni-corrected *P* value < 0.05) were identified from the 480,912 CpGs. To identify age-related methylation, we analyzed the relationship between methylation levels of individual CpGs and patient’s age in 42 normal lung tissues. Thirty-two CpGs (*P* < 1.1E-05) showing significant positive correlation to patient’s age such as cg26354128 (*γ* = 0.64; *P* = 1.86E-06; Fig. 1d) were removed from further data analysis.

### Feature selection for disease classification and the evaluation of proposed models in the TCGA lung cancer data

To perform the selection of features from 4694 CpGs and construct a robust classification model, we used a GO-based approach rather than a gene-based approach because biological processes change as a function of gradual accumulation of alterations in multiple genes. In addition, each biological pathway contributes to tumorigenesis to a different degree, though genes annotated with the same GO term share common biological functions and processes. Four thousand six hundred and ninety-four CpGs were annotated using gene set enrichment analysis. Among 46 candidate GO terms (Bonferroni *P* < 1.0E-5), three GO terms (i.e., transcription regulation, cell-cell signaling, and cell-cell adhesion; Bonferroni *P* = 3.7E-24, 4.4E-8, and 2.9E-6, respectively) were selected due to high sensitivity and specificity of lung cancer classification. Ten CpGs (Additional file 1) of the 4694 CpGs were finally selected for disease classification. Genes including the 10 CpGs were classified into three GO categories: (i) transcription (*HOXA9*, *SOX17*, *ZNF154*, *HOXD13*), (ii) cell signaling (*HBP1*, *SFRP1*, *VIPR2*), and (iii) adhesion (*PCDH17*, ITGA5, *CD34*). Methylation levels of the 10 CpGs were compared according to histology (Fig. 1e). HBP1 and ZNF154 showed higher methylation in other cell types compared to adenocarcinoma and squamous cell carcinoma, but model building was performed without stratification of data according to histology due to the lack of significant interaction between methylation levels and histology (data not shown).

**Fig. 2:**
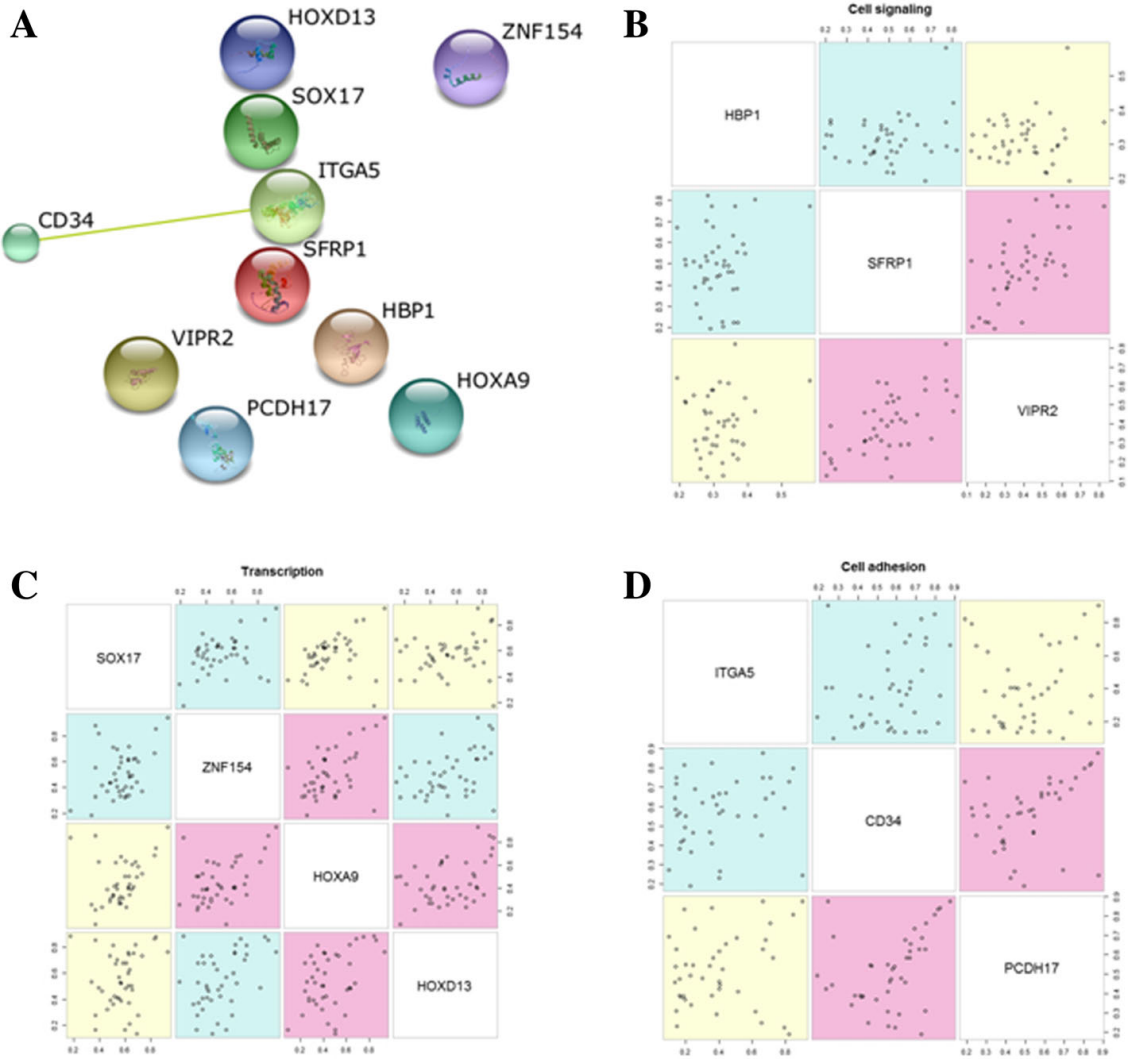
Relationship among methylation levels of 10 genes. **a** Protein-protein interaction was determined by the STRING protein interaction database (https://string-db.org/). **b**–**d** Correlation among methylation levels of genes for each of the three GO categories (transcription, cell signaling, and cell adhesion) was analyzed using Spearman’s correlation coefficient in 42 lung cancers. Magenta color indicates *P* < 0.05

**Fig. 3:**
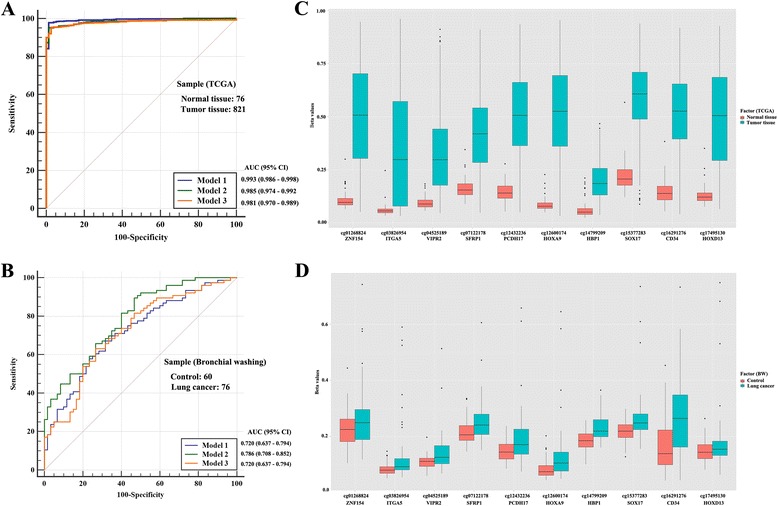
Evaluation of prediction performance of models. The prediction performance of the proposed model using the 10 CpGs was tested in 897 TCGA primary lung tissues (**a**, **b**) and in 136 bronchial washing samples (**c**, **d**). **a**, **c** The area under curve (AUC) of the receiver operating characteristic (ROC) in prediction of lung cancer using three models was 0.981–0.993 for 897 TCGA primary lung tissues and 0.720–0.786 for 136 bronchial washings. ROC curve was produced using MedCalc software. **b**, **d** The methylation levels of 10 CpGs selected in this study were compared between the 821 TCGA primary lung tumor tissues and 76 normal tissues (**b**) and between 76 lung cancer patients and 60 controls (**d**). All CpGs in the TCGA data were found to be highly methylated compared to the matched normal tissues (*P* < 0.0001; Wilcoxon rank-sum test). Although most CpGs in the bronchial washing samples did show a statistically significant difference in the methylation levels between 76 lung cancer patients and 60 controls, the difference was small compared to that in the TCGA data. The *X*- and *Y*-axes indicate CpG numbers and β-values from 450K array, respectively

To build a parsimonious model for lung cancer classification, we analyzed the relationship among 10 genes. Protein-protein interactions were found between CD34 and ITGA5 in STRING (Fig. 2a). In addition, statistically significant correlations within each GO categories were found between methylation levels of ZNF154 and HOXA9 (Fig. 2b), SFRP1 and VIPR2 (Fig. 2c), and CD34 and PCDH17 (Fig. 2d). Significantly correlated CpGs within each individual GO term were not included simultaneously in a model for disease classification. Three subsets of features were chosen for disease classification using logistic regression analysis (Table 2). Prediction performance of three proposed models was tested in 897 TCGA primary lung tissues; the models were able to classify the lung tissues (821 tumor tissues and 76 normal tissues) with a sensitivity of 95.0~97.8% and a 97.4~98.7% of specificity (Fig. 3a).

**Table 2:** The best classification models for lung cancer identified using likelihood ratio test

Model	Model covariates	− 2logL	*P* value
1	HOXA9, SOX17, SFRP1, PCDH17, ITGA5	0.593	< 0.0001
2	SOX17, ZNF154, SFRP1, PCDH17, ITGA5	0.336	< 0.0001
3	SOX17, ZNF154, HBP1, SFRP1, PCDH17, ITGA5	0.230	< 0.0001

### Methylation levels of CpGs in bronchial washing are different from those in lung tumor tissues

To test if diagnostic panels of the 10 CpGs selected from tumor tissues are applicable to bronchial washing, we measured the methylation levels of 136 bronchial washing samples using the 450K array. The 450K data from 42 lung tumor and matched normal tissue samples were used as a training dataset. The area under curves (AUC) of the ROC for prediction of lung cancer using the models was 0.720~0.786 (Fig. 3b). Lung cancer in bronchial washing specimens was predicted with 63.2~89.8% sensitivity and 53.3~73.3% specificity.

To understand factors responsible for the low accuracy of lung cancer prediction in bronchial washing specimens compared to TCGA, we compared methylation levels of the 10 CpGs in 897 TCGA primary lung tissues (Fig. 3c) and in bronchial washing samples from 76 lung cancers and 60 hospital-based controls (Fig. 3d). All 10 CpGs were significantly methylated in the 821 tumor tissues compared to the 76 normal tissues (*P* < 0.0001; Fig. 3c). For bronchial washing samples, nine CpGs except cg17496130 (*P* = 0.10) showed significantly different methylation levels between 76 lung cancer patients and 60 healthy individuals, but the difference was not high compared to TCGA tissue samples (Fig. 3d). Based on these observations, it is likely that a diagnostic panel of methylated CpGs selected from tumor tissues may not allow for accurate prediction of lung cancer in bronchial washing specimens.

### Methylation patterns in bronchial biopsy are comparable to those in lung tumor tissue

**Fig. 4:**
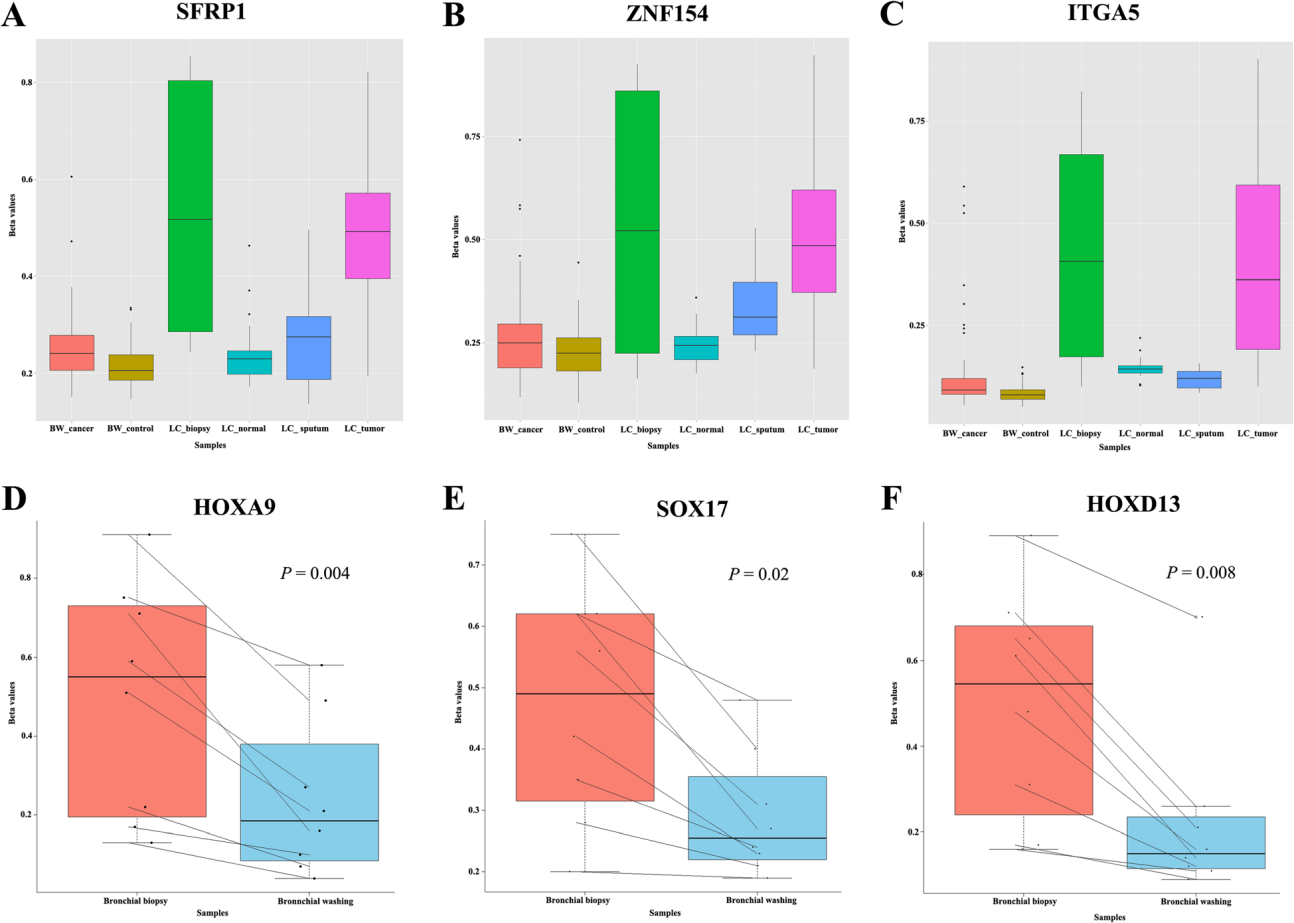
Comparison of methylation levels of CpGs across different kinds of samples. **a**–**c** Methylation levels of three CpGs (cg07122178, cg01268824, and cg03826594) at *SFRP1*, *ZNF154*, and *ITGA5* genes were compared in bronchial washing samples from 76 lung cancer patients (BW_cancer) and 60 healthy individuals (BW_control), bronchial biopsies from 8 lung cancer patients (LC_biopsy), 42 lung tumor (LC_tumor) and matched normal tissues (LC_normal), and in sputum from 12 lung cancer patients (LC_sputum). **d**–**f** Methylation levels of three CpGs (cg12600174, cg15377283, and cg17495130) at *HOXA9*, *SOX17*, and *HOXD13* genes were compared using parallel coordinate plots between paired bronchial biopsy and bronchial washing specimens from eight lung cancer patients. Methylation levels were significantly higher in bronchial biopsy than in bronchial washing samples (*P* < 0.05, Wilcoxon signed-rank test). *Y*-axis indicates β-values from the 450K array

To test if bronchial biopsy specimens can be used as a surrogate for DNA methylation analysis in lung cancer, we compared the methylation levels of the 10 CpGs in eight bronchial biopsy specimens from lung cancer patients (Fig. 4). The methylation levels of the 10 CpGs in lung tumor tissues were not significantly different from those in bronchial biopsy from lung cancer patients (*P* > 0.05, Wilcoxon rank-sum test; Figs. 4a–c; Additional file 2). The methylation levels of the 10 CpGs were further compared between paired bronchial biopsies and bronchial washings from eight lung cancer patients. The methylation levels of the 10 CpGs, such as the three CpGs in Fig. 4d, e, f, were found to be higher in bronchial biopsy than in bronchial washing. Furthermore, the difference was statistically significant (*P* < 0.05, Wilcoxon signed-rank test).

**Fig. 5:**
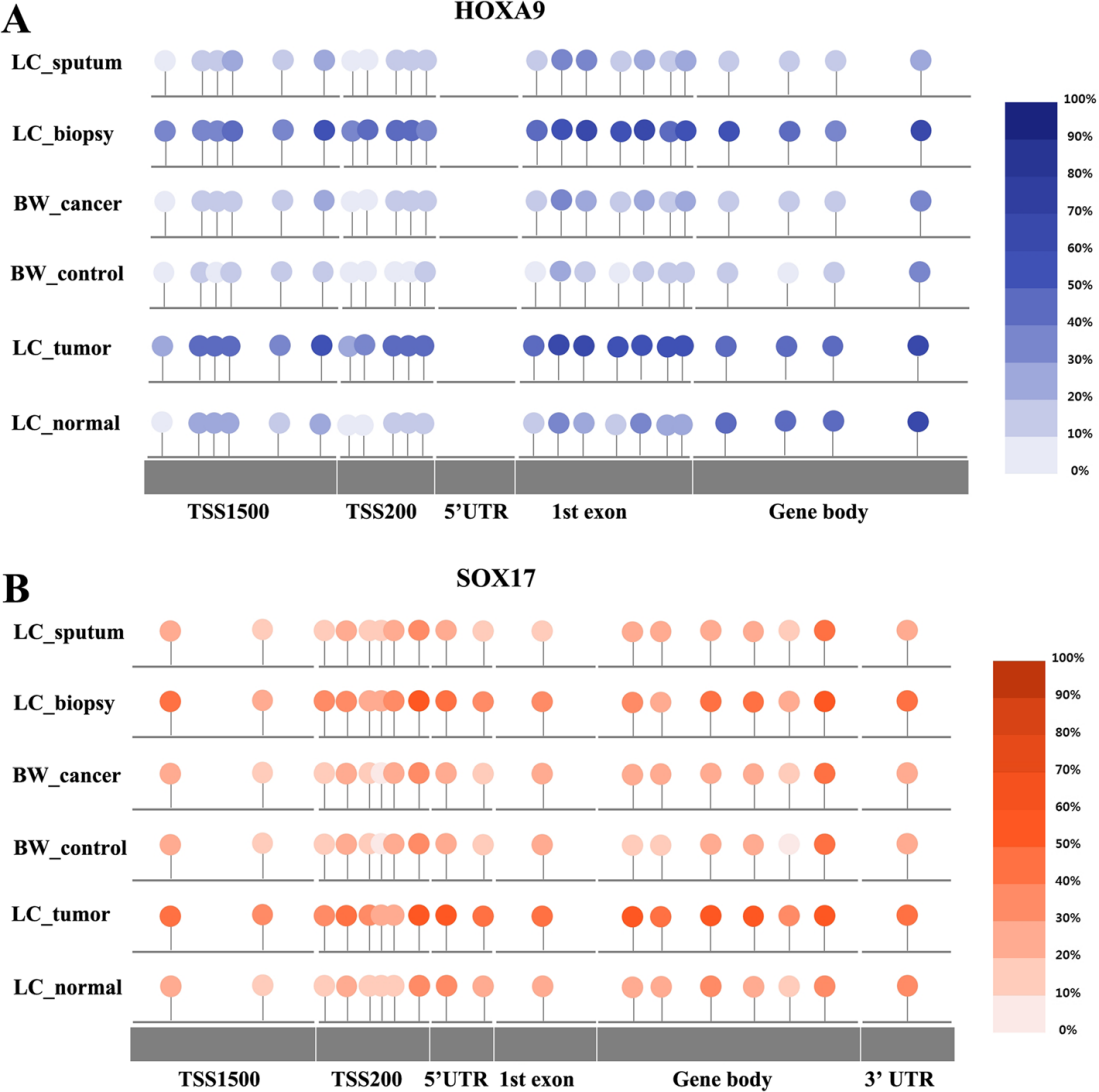
Methylation levels of multiple CpGs in *HOXA9* and *SOX17* genes. Methylation levels of 21 CpGs in *HOXA9* gene (**a**) and of 18 CpGs in *SOX17* gene (**b**) were analyzed across different kinds of samples. Colors indicate average methylation levels of individual CpGs in sputum from 12 lung cancer patients (LC_sputum), bronchial biopsies from 8 lung cancer patients (LC_biopsy), bronchial washing samples from 76 lung cancer patients (BW_cancer) and 60 healthy individuals (BW_control), and in 42 lung tumor (LC_tumor) and matched normal tissues (LC_normal)

Finally, we analyzed methylation levels of multiple CpGs within the genes selected in this study. The methylation levels of *HOX9* (Fig. 5a) and *SOX17* (Fig. 5b) are shown as representative examples. Twenty-one CpGs in *HOXA9* and 18 CpGs in *SOX17* were analyzed in sputum, bronchial washing, bronchial biopsy, and lung tissue specimens. Although CpGs located in the gene body showed methylation in lung normal tissues, other CpGs in TSS1500, TSS200, 5’ UTR, and the first exon were similarly methylated in bronchial biopsy from lung cancer patients as well as in surgically resected tumor tissues. These data suggest that bronchial biopsy specimen may be used as a surrogate for DNA methylation analysis in lung cancer.

## Discussion

Although several potential biomarkers that are associated with lung cancer have been identified in sputum and bronchial washing [16, 17], few biomarkers have been sufficiently validated for use in clinical applications. Several groups have reported that abnormal methylation observed in tumor tissues is also found in bronchial washing and sputum samples [18, 19], suggesting that biomarkers selected from lung tumor tissues may be applicable to bronchial washing and sputum samples. However, the methylation levels of the 10 CpGs identified from 42 lung tumor tissues in this study were generally very low in bronchial washing samples from lung cancer patients (Fig 4c). In addition, most of the highly methylated CpGs in lung tumor tissues did not show significant methylation in exfoliated bronchial epithelial cells from bronchial washing, and a small number of CpGs showed a statistically significant methylation in bronchial washing (data not shown). Accordingly, it is likely that lung tumor tissues may have different patterns of DNA methylation compared to bronchial washing and sputum.

It is not clear what is responsible for different methylation profiling between lung tumor tissues and exfoliated bronchial epithelial cells from lung cancer patients. The low methylation levels of the CpGs in bronchial washing and sputum samples compared to tumor tissues might result from contamination by normal bronchial epithelial cells or from an inadequacy of bronchial washing and sputum samples. In addition, methylation levels in sputum samples may be affected by the contamination of inflammatory cells such as neutrophils and macrophages [20]. Bronchial washing and sputum samples are also known to be inadequate for the identification of centrally located tumors such as small cell lung cancer and squamous cell carcinoma and in peripherally located tumors, respectively. Accordingly, standardization of processing protocols and bronchial epithelial cell enrichment is required for a greater yield of bronchial epithelial cells, and sample collection using a laser-induced fluorescence bronchoscopy may reduce normal cell contamination. Another possibility is that CpG hypermethylation of genes that are relevant to the progression from hyperplasia and dysplasia to invasive or metastatic lung cancer may not be detectable in exfoliated bronchial epithelial cells. For example, the methylation levels at cg12600174 (Fig. 5a) and cg15377283 (Fig. 5b) at the promoter regions of *HOXA9* and *SOX17* genes in lung tumor tissues, respectively, were found to be similar to those in bronchial biopsy, but not in bronchial washing and sputum. Several studies suggest that HOXA9 [21–23] and SOX17 [24–27] are involved in tumor progression. In addition, HBP1 and SFRP1 included in the models (Additional file 1) are known to function as suppressors of cancer progression by suppressing β-catenin transactivation [28], and cell adhesion-related genes are involved in tumor invasion or metastasis.

Although the number of bronchial biopsy specimens required to provide accurate molecular analysis has not been defined, we extracted genomic DNA from one specimen for the analysis of methylation using the 450K array. A minimum of 2 μg of genomic DNA was extracted from all bronchial biopsy specimens. The amount of tumor present in bronchial biopsy specimens is relatively low; the mean percentage area of tumor in a biopsy specimen was 33.4%, and tumor was found in fewer than half of cancer cases [29]. We did not measure the percentage of tumor cells in our bronchial biopsy specimens, but methylation levels of CpGs from bronchial biopsy were comparable to those from surgically resected lung tumor tissues. Although we collected samples through fiberoptic bronchoscopy, new systems such as endobronchial ultrasonography using a guided sheath (EBUS-GS) and electromagnetic navigation (ENB) will provide more appropriate bronchial biopsy specimens for molecular analysis. In addition, advances in technology will lead to improved yields with fewer complications even in the peripheral regions of bronchus.

We used a GO-based approach instead of a gene-based approach as a gene can be involved in multiple different biological processes. Besides, a biological pathway is usually composed of the negative or positive action of multiple genes and contributes to tumorigenesis to a different degree. We identified relevant biological pathways using gene-set enrichment analysis and deleted CpGs that were significantly correlated in the same pathway in order to make our model more parsimonious because simultaneous alterations in correlated genes in a pathway are usually redundant in terms of the alteration of that pathway. In this study, supervised machine learning algorithms, including support vector machine [30], decision tree, K-Nearest Neighbor (K-NN), random forest [31], and artificial neural network [32], were utilized for the classification of lung cancer; however, their classification performance was not better than a logistic regression analysis (data not shown). Three models using logistic regression analysis showed similar sensitivity and specificity in classifying lung cancer, suggesting that multiple models may be possible and result from lung cancer heterogeneity.

This study was limited by several factors. Above all, this study was conducted in a small number of bronchial biopsy specimens, especially a low number of paired biopsy and lung resections. In addition, this study could not cover the complex heterogeneity of lung cancer and compare methylation levels of discovered CpGs in paired samples, including tumor tissue, bronchial biopsy, sputum, and bronchial washing due to the shortage of samples. Accordingly, rare-type tumors and paired samples need to be further analyzed by prospective large-scale studies.

## Conclusions

Precision medicine using epigenetic priming before cytotoxic or targeted therapy requires methylation statuses of predictive response biomarkers in lung cancer. The present study suggests that bronchial biopsy specimen may be used as a surrogate for DNA methylation analysis in inoperable lung cancer patient.

## Additional files

Additional file 1: Differentially methylated regions (DMRs). (DOCX 14 kb)

Additional file 2: Methylation levels of seven CpGs across different kinds of samples. Methylation levels of seven CpGs at *PCDH7* (A), *VIPR2* (B), *HBP1* (C), *CD34* (D), *HOXA9* (E), *SOX17* (F), and *HOXD13* (G) were compared among six different kinds of samples: bronchial washing samples from 76 lung cancer patients (BW_cancer), 60 healthy individuals (BW_control), bronchial biopsies from 8 lung cancer patients (LC_biopsy), 42 lung tumor (LC_tumor) and matched normal tissues (LC_normal), and sputum from 12 lung cancer patients (LC_sputum). *Y*-axis indicates β-values from the 450K array. (TIFF 3249 kb)

## Abbreviations

DMRs: Differentially methylated regions
DNMT: DNA methyltransferase
EBUS-GS: Endobronchial ultrasonography using a guided sheath
GO: Gene ontology
HDAC: Histone deacetylase
ROC: Receiver operating characteristic
TCGA: The Cancer Genome Atlas
TNM: Tumor-node-metastasis
