# Supplementary material for: Bronchial biopsy specimen as a surrogate for DNA methylation analysis in inoperable lung cancer
Source: Clin Epigenetics. 2017 Dec 20;9:131. doi: 10.1186/s13148-017-0432-5 (PMC5738682; doi:10.1186/s13148-017-0432-5)
Supplement: Supplementary file 1 — Differentially methylated regions (DMRs). (DOCX 14 kb) [file 13148_2017_432_MOESM1_ESM.docx]

**Additional file 1: Differentially methylated regions (DMRs)**

| TargetID | UCSC_REFGENE_NAME | UCSC_CPG_ISLANDS_NAME | UCSC_REFGENE_GROUP | RELATION_TO_  UCSC_CPG_ISLAND | MAPINFO |
| --- | --- | --- | --- | --- | --- |
| cg12600174 | HOXA9 | chr7:27203915-27206462 | TSS200 | Island | 27205230 |
| cg15377283 | SOX17 | chr8:55370170-55372525 | Body | Island | 55371369 |
| cg01268824 | ZNF154 | chr19:58220189-58220517 | TSS1500 | S_Shore | 58220818 |
| cg17495130 | HOXD13 | chr2:176962179-176962487 | 3' UTR | N_Shore | 176960255 |
| cg14799209 | HBP1 | chr7:106809461-106809815 | 5' UTR | Island | 106809543 |
| cg07122178 | SFRP1 | chr8:41165852-41167140 | TSS1500 | S_Shore | 41168481 |
| cg04525189 | VIPR2 | chr7:158936507-158938492 | Body | Island | 158936508 |
| cg12432236 | PCDH17 | chr13:58206526-58208930 | 1st Exon | S_Shore | 58209133 |
| cg03826594 | ITGA5 | chr12:54811981-54812202 | Body | Island | 54812085 |
| cg16291276 | CD34 | chr1:208084098-208084513 | Body | N-Shore | 208084071 |

Abbreviations: UCSC_REFGENE_NAME, Gene name (UCSC);

UCSC_CPG_ISLANDS_NAME, CpG island name (UCSC);

UCSC_REFGENE_GROUP, Gene region feature category (UCSC);

Relation_to_UCSC_CpG_Island, Relationship to Canonical CpG Island, Shores - 0-2 kb from CpG island; Shelves - 2-4 kb from CpG island.

MAPINFO, Coordinates - genome build 37
