# Supplementary figures and images for: Bronchial biopsy specimen as a surrogate for DNA methylation analysis in inoperable lung cancer
Source: Clin Epigenetics. 2017 Dec 20;9:131. doi: 10.1186/s13148-017-0432-5 (PMC5738682; doi:10.1186/s13148-017-0432-5)

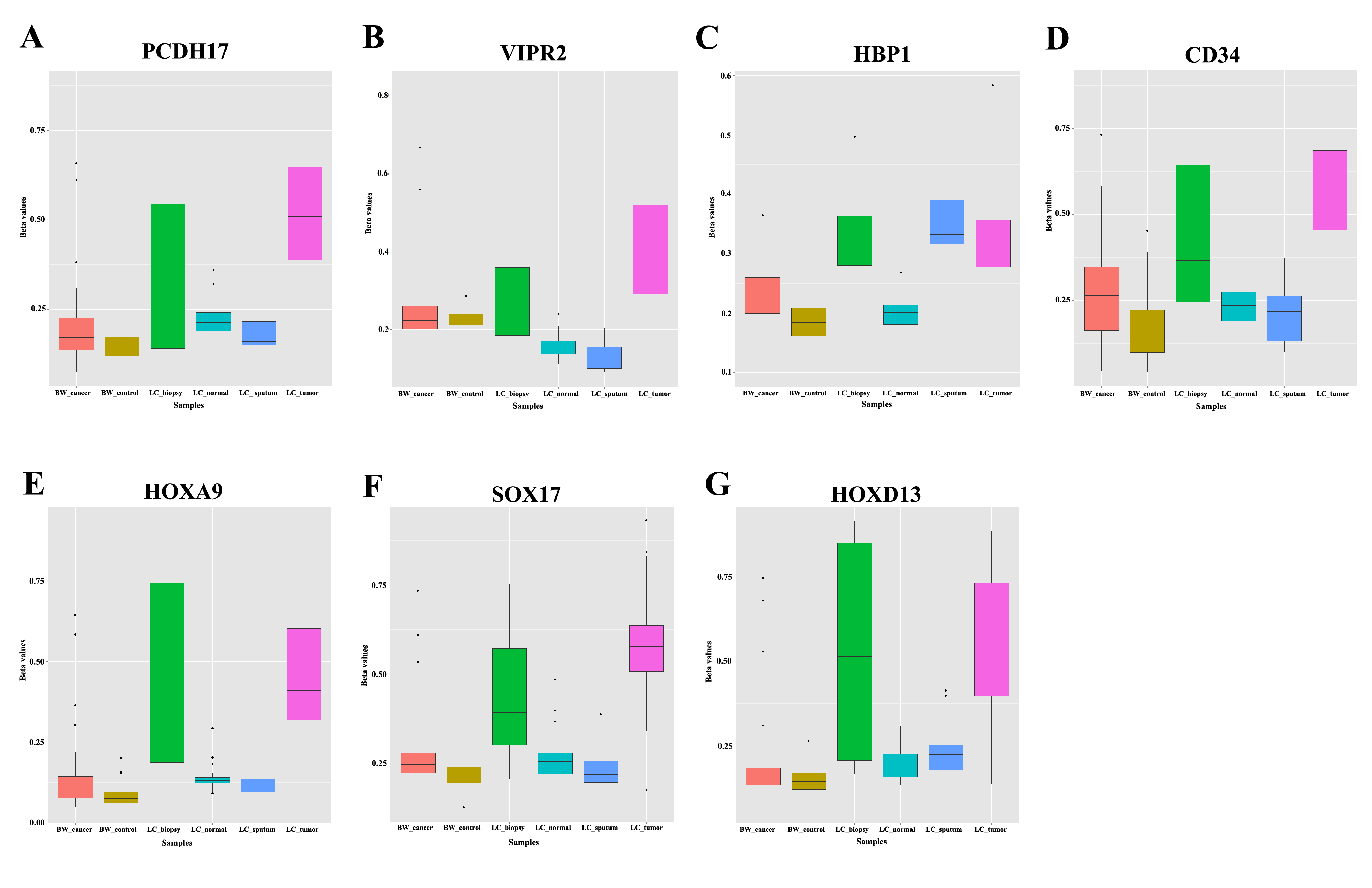

Supplement: Supplementary file 2 — Methylation levels of seven CpGs across different kinds of samples. Methylation levels of seven CpGs at PCDH7 (A), VIPR2 (B), HBP1 (C), CD34 (D), HOXA9 (E), SOX17 (F), and HOXD13 (G) were compared among six different kinds of samples: bronchial washing samples from 76 lung cancer patients (BW_cancer), 60 healthy individuals (BW_control), bronchial biopsies from 8 lung cancer patients (LC_biopsy), 42 lung tumor (LC_tumor) and matched normal tissues (LC_normal), and sputum from 12 lung cancer patients (LC_sputum). Y-axis indicates β-values from the 450K array. (TIFF 3249 kb) [file 13148_2017_432_MOESM2_ESM.tif]
